# Emphysematous gastritis with portal venous gas: conservative management in a high-risk patient

**DOI:** 10.1093/jscr/rjag089

**Published:** 2026-02-27

**Authors:** Sandeepa Dadigamuwage, Vimarshini Samarakoon, Emma Horrocks

**Affiliations:** Colorectal Surgery Department, University Hospitals Plymouth NHS Trust, Derriford Road, Crownhill, Plymouth, Devon, PL6 8DH, United Kingdom; Torrington Cardiac Intensive Care Unit, University Hospitals Plymouth NHS Trust, Derriford Road, Crownhill, Plymouth, Devon, PL6 8DH, United Kingdom; Colorectal Surgery Department, University Hospitals Plymouth NHS Trust, Derriford Road, Crownhill, Plymouth, Devon, PL6 8DH, United Kingdom

**Keywords:** emphysematous gastritis, portal venous gas, conservative management, frailty, pressure ulcer

## Abstract

Emphysematous gastritis is a rare, often fatal condition characterized by gas within the gastric wall, usually in patients with significant comorbidities. We describe a 75-year-old man with incomplete tetraparesis, chronic respiratory failure and type 2 diabetes who presented with abdominal pain, vomiting and hematemesis. Initial computed tomography (CT) was reported as non-obstructive small-bowel dilatation with possible gallstone ileus, but subspecialty radiology review identified intramural gastric gas with portal venous gas consistent with emphysematous gastritis. He was managed non-operatively with nasogastric decompression, intravenous broad-spectrum antibiotics and proton-pump inhibition within a ward-based ceiling of care. Repeat CT on day 10 showed complete radiological resolution. Despite this, he later developed sepsis from a large necrotic pressure ulcer and died after a multidisciplinary decision for palliative care. This case supports conservative management in selected high-risk patients and emphasizes early, meticulous pressure-area assessment in immobile surgical admissions.

## Introduction

Emphysematous gastritis is a rare but life-threatening form of gastritis, characterized by intramural gastric gas from infection with gas-forming organisms in patients with diabetes, immunosuppression or other severe comorbidities [1, 2]. Mortality may reach 50–60% [1, 2]. It must be distinguished from benign gastric emphysema, which has a far more favorable course [3]. There is no consensus on management: earlier reports describe emergency gastrectomy, whereas more recent series support conservative treatment with bowel rest, broad-spectrum antibiotics and close radiological monitoring in the absence of perforation or hemodynamic instability [1, 2, 4, 5]. We report a frail tetraparetic patient with emphysematous gastritis and portal venous gas successfully managed non-operatively, whose eventual death was due to sepsis from a late-recognized pressure ulcer.

## Case report

A 75-year-old man presented with a two-day history of worsening upper abdominal pain, nausea and multiple episodes of vomiting, with no stool or flatus and one episode of hematemesis. His past medical history included incomplete C4 tetraparesis after traumatic spinal injury, atypical bipolar disorder, type 2 diabetes mellitus, ischemic heart disease, chronic type 2 respiratory failure, obstructive sleep apnea, a left clinoid/sphenoid meningioma under neurosurgical follow-up, previous pulmonary embolism and a long-term suprapubic catheter. Usual medications were baclofen, gabapentin, tizanidine, olanzapine, metformin and rosuvastatin. He was hemodynamically stable, with a distended but soft abdomen and upper abdominal discomfort without peritonism. Digital rectal examination showed hard stool but no masses or blood. Initial hematological investigations and blood gas analysis results are summarized in Tables 1 and 2.

**Table 1:** Hematological investigation results on admission.

Parameter	Value on admission	Reference range
eGFR	89	>90 ml/min/1.73 m^2^
Hemoglobin (g/L)	151	130–175 g/L
White Cell Count	15.6	3.6–9.2 x 109/L
Neutrophils	12.0	1.7–6.2 x 109/L
Platelets	209	150–450 x 109/L
C-reactive protein	4	0.1–5 mg/L
Urea	6.8	2.5–7.8 mmol/L
Creatinine	68	64–104 μmol/L
Sodium	138	133–146 mmol/L
Potassium	4.7	3.5–5.3 mmol/L
Alkaline Phosphatase	52	30–130 IU/L
Bilirubin	4	1–20 μmol/L
Alanine Transaminase	20	1–55 IU/L
Activated Partial Thromboplastin Time	46.2	24–34 seconds
International Normalization Ratio	1.1	N/A
Prothrombin Time	16.6	12.5–15.5 seconds

**Table 2:** Blood gas analysis on admission.

Parameter	Results	Reference range
pH	7.38	7.35–7.45
PaO₂	7.17	10–13 kPa
PaCO₂	7.40	4.7–6.0 kPa
Bicarbonate	33.1	22–26 mmol/L
Lactate	3.1	0.5–2.0 mmol/L

**Abbreviations: pH**, potential of hydrogen; **PaO₂**, partial pressure of arterial oxygen; **PaCO₂**, partial pressure of arterial carbon dioxide; **SaO₂**, arterial oxygen saturation.

Contrast-enhanced computed tomography (CT) of the abdomen and pelvis reported dilated small-bowel loops without a definite transition point, no large-bowel obstruction and appearances thought to represent gallstone ileus, with subcapsular hepatic gas interpreted as pneumobilia. He was managed as presumed gallstone ileus with nasogastric decompression, nil by mouth, intravenous fluids and intravenous amoxicillin–clavulanate plus metronidazole. Overnight he desaturated to 68% in the context of known obstructive sleep apnea and chronic respiratory failure. After a documented capacity assessment he refused continuous positive airway pressure or non-invasive ventilation. His treatment escalation plan was revised to ward-based care with do-not-attempt cardiopulmonary resuscitation, and he was not considered for intensive care unit admission.

**Figure 1:**
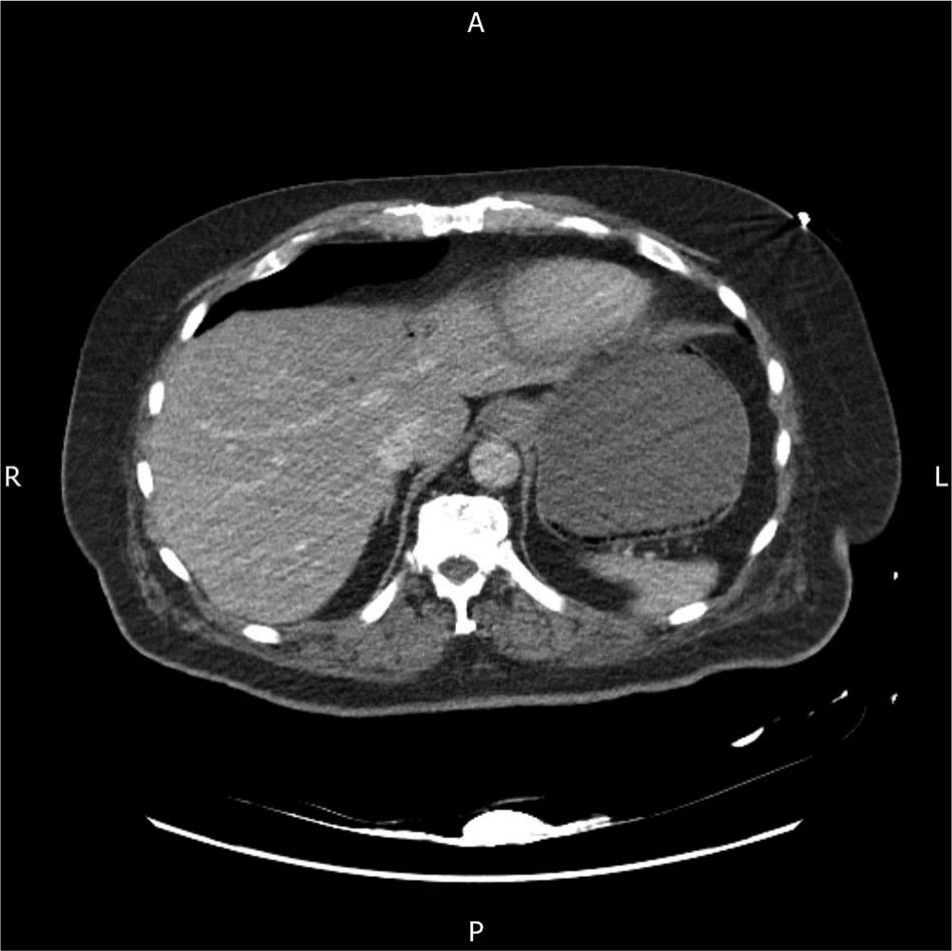
Axial contrast-enhanced CT abdomen on admission showing mottled intramural gas within the gastric wall and associated hepatic portal venous gas in the left lobe, in keeping with emphysematous gastritis.

Following discussion with the referring team, the CT images were re-reviewed by a gastrointestinal radiologist. An addendum reported portal venous gas, gas within the left and right gastroepiploic vessels and submucosal gas within the stomach, consistent with acute gastric distension and emphysematous gastritis in a diabetic patient (Fig. 1). Piperacillin–tazobactam replaced amoxicillin–clavulanate to broaden gram-negative and anaerobic cover, and intravenous proton-pump inhibitor therapy was continued. Chest radiography showed left lower-lobe consolidation and he was treated for aspiration pneumonia. The oesophago-gastric team, noting his stability, frailty, high anesthetic risk and lack of peritonism or perforation, advised conservative management with nasogastric decompression and antibiotics, and outpatient oesophagogastroduodenoscopy once recovered.

On day 10 a planned repeat CT demonstrated complete resolution of portal venous gas, near-complete resolution of intramural gastric gas and decompressed small bowel, with only mild sigmoid distension and no evidence of mechanical obstruction or volvulus. His abdominal pain and vomiting had improved, but he continued to spike fevers while on broad-spectrum antibiotics. Blood cultures and viral swabs were negative. A detailed pressure-area assessment in a lateral position revealed a large necrotic gluteal pressure ulcer on the right buttock, presumed to be the source of sepsis. Examination under anesthesia showed a cavity measuring approximately 9.5 × 6.5 cm with a depth of 4.5 cm; muscle and bone were not involved. Surgical debridement and washout were performed, and negative-pressure wound therapy was applied by the tissue-viability team. Antibiotics were escalated to include clindamycin and teicoplanin for soft-tissue coverage.

In the following days he developed fluctuating consciousness, recurrent desaturation and a persistent inflammatory response despite treatment. A further CT scan excluded sacral osteomyelitis. After several medical emergency team calls and multidisciplinary discussions involving surgical, medical and palliative care teams with his next of kin, further escalation was deemed not in his best interests. He was transitioned to an end-of-life care plan and died 30 days after admission.

## Discussion

This case highlights key features of emphysematous gastritis and its care in frail surgical patients. Emphysematous gastritis is rare, but more frequently recognized with modern CT [1, 2, 6, 7]. It results from mucosal injury with invasion by gas-forming organisms, often in the setting of diabetes or immunosuppression [1, 2, 6, 7]. Affected patients are usually systemically unwell, unlike those with benign gastric emphysema [3, 6], and imaging typically shows mottled intramural gas with wall thickening, sometimes with portal venous gas as in this case [3, 6, 7].

Management has shifted from routine emergency gastrectomy towards selective surgery [1, 2]. Recent reports describe successful conservative treatment with bowel rest, nasogastric decompression, proton-pump inhibition and broad-spectrum antibiotics when there is no perforation, peritonitis or hemodynamic instability [4, 5, 7]. Our patient, despite multiple comorbidities, achieved complete radiological and symptomatic resolution on day-10 CT, supporting an initial non-operative approach in carefully selected, stable patients whose surgical risk is prohibitive [4, 5, 7].

The case underlines the importance of good communication with radiology. The first CT was reported as small-bowel dilatation with possible gallstone ileus, and the critical findings of portal venous and intramural gastric gas were only identified on expert re-review. Early multidisciplinary discussion of equivocal scans may help avoid diagnostic delay in similar presentations.

Finally, the patient’s death resulted not from the gastric pathology but from sepsis due to a large pressure ulcer detected late in his admission. Patients with tetraparesis, chronic respiratory failure and prolonged bed rest are at high risk of pressure injuries [8]. Early, thorough skin inspection with repositioning to visualize sacral and buttock areas, repeated at regular intervals, and prompt escalation to tissue-viability services at the first sign of damage are essential to reduce the risk of deep tissue necrosis, sepsis and extensive debridement [8]. In conclusion, emphysematous gastritis should be considered in frail, multimorbid patients with severe abdominal pain and intramural gastric gas on CT, particularly when portal venous gas is present, and can be managed conservatively in selected cases, provided equal attention is paid to basic pressure care in immobile surgical patients.
